# A set of *Yarrowia lipolytica* CRISPR/Cas9 vectors for exploiting wild-type strain diversity

**DOI:** 10.1007/s10529-020-02805-4

**Published:** 2020-01-23

**Authors:** Macarena Larroude, Heykel Trabelsi, Jean-Marc Nicaud, Tristan Rossignol

**Affiliations:** Université Paris-Saclay, INRAE, Micalis Institute, 78350 Jouy-en-Josas, AgroParisTech France

**Keywords:** CRISPR/Cas9, *Yarrowia lipolytica*, Golden Gate, GSY1, Synthetic biology

## Abstract

**Objectives:**

The construction and validation of a set of *Yarrowia lipolytica* CRISPR/Cas9 vectors containing six different markers that allows virtually any genetic background to be edited, including those of wild-type strains.

**Results:**

Using the Golden Gate method, we assembled a set of six CRISPR/Cas9 vectors, each containing a different selection marker, to be used for editing the genome of the industrial yeast *Y. lipolytica*. This vector set is available via Addgene. Any guide RNA (gRNA) sequence can be easily and rapidly introduced in any of these vectors using Golden Gate assembly. We successfully edited six different genes in a variety of genetic backgrounds, including those of wild-type strains, with five of the six vectors. Use of these vectors strongly improved homologous recombination and cassette integration at a specific locus.

**Conclusions:**

We have created a versatile and modular set of CRISPR/Cas9 vectors that will allow any *Y. lipolytica* strain to be rapidly edited; this tool will facilitate experimentation with any prototroph wild-type strains displaying interesting features.

**Electronic supplementary material:**

The online version of this article (10.1007/s10529-020-02805-4) contains supplementary material, which is available to authorized users.

## Introduction

*Yarrowia lipolytica* is widely used as a microbial cell factory chassis in the development of industrial applications aiming to produce fatty acids, organic acids, or enzymes (Nicaud 2012; Madzak 2015; Ledesma-Amaro and Nicaud 2016). Although many engineering tools are now available (Larroude et al. 2018), the high rate of non-homologous end joining (NHEJ) impairs the efficiency of targeted genome modification. Moreover, limitations related to selection markers and the need to recycle them make engineering efforts even more time-consuming, and it can be challenging to generate highly modified strains with traits that correspond to the demands of industrial processes. CRISPR/Cas9 technology is being continually refined, and it was rapidly implemented in *Saccharomyces cerevisiae* (DiCarlo et al. 2013). It has also been successfully used in *Y. lipolytica*, with the aim of overcoming the aforementioned limitations and accelerating engineering cycles.

Several CRISPR/Cas9 systems dedicated to *Y. lipolytica* have recently been described. Both an RNA polymerase II (Pol II) transcription system and RNA polymerase III (Pol III) elements have been set up. Gao et al. (Gao et al. 2016) developed a Pol II gene disruption system that has successfully yielded single to triple mutations in a single step. Schwartz et al. (Schwartz et al. 2016) used a Pol III system and developed an efficient synthetic Pol III-tRNA hybrid promoter for gene disruption. In addition, a guide RNA (gRNA) system involving the expression of orthogonal T7 polymerase was exported to *Y. lipolytica*; it was based on the pCRISPRyl vector of Schwartz et al. (Morse et al. 2018). Structure–function relationships study and methodological research focused on single point mutations has also been described (Borsenberger et al. 2018). In addition, alternative systems, such as using defective Cas9 (CRISPRi) to inhibit expression, CRISPRa to activate gene expression, and the dual CRISPR/Cas9 system to excise and integrate genes have been shown to function in *Y. lipolytica* (Schwartz et al. 2017a, 2018; Gao et al. 2018).

It is now well established that the CRISPR/Cas9 system functions properly in *Y. lipolytica*, and modular and robust tools are needed for this system to become the standard for editing the genome of this yeast species. Such tools are particularly lacking when it comes to engineering the diversity of wild-type strains. These strains are never used as there are no markers available for them, which hinders metabolic engineering. To date, researchers have mainly concentrated on “laboratory” strains for which auxotrophic markers are already available. However, it is now well known that wild-type isolates display a wide range of traits when it comes to producing lipids, citric acids, or polyols, for example (Egermeier et al. 2017; Quarterman et al. 2017; Carsanba et al. 2019); in these experiments, some of the isolates strongly outperformed the standard laboratory strains. Wild-type strains may also respond very differently to environmental conditions and control parameters (Egermeier et al. 2017).

Having selectable markers is crucial to the success of genome editing technologies. In *Y. lipolytica*, such markers were initially based on leucine (LEU2), uracil (URA3), lysine (LYS5) and adenine (ADE2) auxotrophies (Barth and Gaillardin 1996). The first dominant markers to be developed relied on the expression of the *E. coli hph* gene, which confers antibiotic resistance to hygromycin B (Barth and Gaillardin 1996). Additional markers have been developed more recently. They are based on the *Streptomyces noursei Nat1* gene (which provides resistance to nourseothricin) (Kretzschmar et al. 2013); the *Y. lipolytica AHAS* gene (which provides resistance to the herbicide chlorimuron ethyl); the *E. coli guaB* gene (which provides resistance to mycophenolic acid) (Wagner et al. 2018); the *Streptoalloteichus hindustanus ble* gene (which provides resistance to zeocin) (Tsakraklides et al. 2018); and the phosphite dehydrogenase *ptxD* gene from *Pseudomonas stutzeri* (which allows *Y. lipolytica* to grow on potassium phosphite in phosphate-deficient media) (Shaw et al. 2016). Additional markers have been developed that are related to the ability to catabolize carbon sources (hereafter referred to as “catabolic selectable markers” [CSM]). These markers have the advantage of not being involved in essential metabolic pathways. For instance, the *S. cerevisiae SUC2* gene, which encodes invertase, has been used to select transformants on sucrose media (Nicaud et al. 1989). More recently, a novel CSM was developed that is centered on the *EYK1* gene, which encodes an erythrulose kinase (Vandermies et al. 2017). This enzyme participates in an early step in erythritol catabolism and is essential for cell growth on erythritol-based medium (Carly et al. 2017).

Here, we describe the construction and validation of a set of seven CRISPR/Cas9 vectors, which each contain a different selection marker. They allow genome editing within virtually any genetic background and are particularly useful for working with wild-type strains, thanks to the panel of dominant markers they represent. Having access to such tools is especially important when it is necessary to integrate multiple cassettes to generate large pathways, which involves the use of multi-auxotrophic strains. gRNA sequences can easily be cloned into these vectors via the Golden Gate method. Consequently, these replication-based CRISPR/Cas9 vectors can be employed to knock out auxotrophic or CSM genes and thus increase the panel of markers available in any strain.

## Materials and methods

### Strains and media

The *Escherichia coli* and *Y. lipolytica* strains and plasmids used in this study are listed in Table S1. *E. coli* strain DH5α was used for cloning and plasmid propagation. The transformation of chemically competent *E. coli* cells was performed using a heat shock protocol. Cells were grown at 37 °C with constant shaking on 5 ml of LB medium (10 g/L tryptone, 5 g/L yeast extract, and 10 g/L NaCl) that contained ampicillin (100 μg/ml) or kanamycin (50 μg/ml) for plasmid selection. For yeast selection and growth, minimal YNBD medium containing 10 g/L glucose (Sigma), 1.7 g/L yeast nitrogen base (YNBww; Difco), 5.0 g/L NH_4_Cl, and 50 mM phosphate buffer (pH6.8) was used. To meet auxotrophic requirements, uracil (0.1 g/L), lysine (0.8 g/L), and leucine (0.1 g/L) were added to the culture medium when necessary. Erythritol and lysine were used as carbon sources at concentrations of 10 g/L and 0.1 g/L, respectively, to allow the selection of *Δeyk1* and *Δeyd1* mutants; lysine was used as an additional carbon source to boost growth in the absence of glucose, but in a concentration not sufficient for *Δeyk1* and *Δeyd1* to grow in presence of erythritol and absence of glucose. An oleic acid emulsion (0.05%) was used as a carbon source for *Δmfe* selection. Tributyrin YNB medium was utilized for *Δlip2* selection as described in (Pignede et al. 2000). For antibiotic selection, hygromycin (250 μg/ml) or nourseothricin (400 μg/ml) was added to rich YPD medium (20 g/L Bacto Peptone, 10 g/L yeast extract, and 20 g/L glucose). Solid media were prepared by adding 15 g/L agar (Invitrogen) to liquid media.

### Construction of acceptor vectors for gRNA cloning

**Fig. 1:**
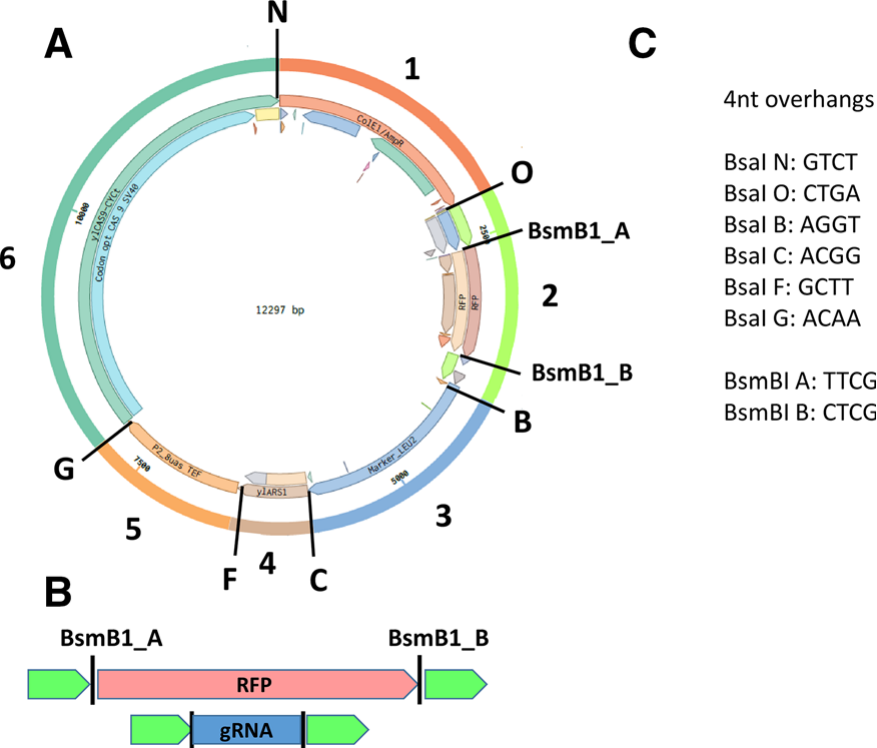
Schematic draw showing an example of a CRISPR/Cas9 acceptor vector assembled via the Golden Gate method. **a** Assembly of the six fragments: the *E. coli* bacterial component (fragment 1); the sgRNA region containing the *E. coli* red fluorescent protein chromophore (RFP) gene flanked by the BsmBI sites (fragment 2); the excisable *Y. lipolytica* marker ylLEU2ex (fragment 3); the *Y. lipolytica* centromeric region ylARS1 (fragment 4); the promoter region P2_8UAS TEF (fragment 5); and the Cas9-SV40 region (fragment 6). **b** Cutting with BsmBI releases the RFP gene and permits the assembly of the 20-nt target sequence at that same location. **c** BsaI and BsmBI overhang sequences

Six fragments were used to assemble the CRISPR/Cas9 acceptor vectors. Fragment 1 was the pSB1A3 bacterial plasmid containing the ampicillin resistance gene and the ColE1 region for selection and propagation in *E. coli*. Fragment 2 was the sgRNA module with the BsmBI recognition sites flanking the red fluorescent protein (RFP) gene, which was used as a chromophore in *E. coli* and which replaced the 20-nt target sequence (Fig. 1b). Fragment 3 was the excisable marker flanked by the I-sceI sites and the LoxP/LoxR. Fragment 4 was a 227-bp portion of the ARS68 sequence described in (Fournier et al. 1991), which allows plasmid replication and segregation in *Y. lipolytica*. Another 105-bp portion of the ARS68 sequence was present at the 3′ end of the sgRNA module and was thus cloned into the vector as part of fragment 2. Fragment 5 was the strong hybrid promoter p8UAS1TEF described by (Celinska et al. 2017), which is used in the expression of the endonuclease. Fragment 6 was the codon-optimized *Streptococcus pyogenes* Cas9-SV40 system including the CYC-t terminator described by Schwartz et al. (Schwartz et al. 2016). The internal BsmBI and BsaI sites were removed from all the fragments, which were then synthetized by Twist Bioscience or GeneMill.

To build fragment 2, the fragment carrying the RFP gene was amplified using primer pair RFP-SfiI-Fw/RFP-SfiI-Rv, and the plasmid GGE029 was employed as a template (Supplementary Table S1). Then, the RFP gene flanked by the SfiI sites was inserted into the sgRNA module (JME4315), which itself included the same SfiI site, giving rise to sgRNA::RFP JME4366 (Table 1).

**Table 1:** Golden Gate fragments available for CRISPR/Cas9 vector assembly

Fragment #	*E. coli*/plasmid name	Region name	Region description	5′ *Bsa*1	3′ *Bsa*1	Reference/source
1	GGE124	AmpR-ORI1	Bacterial vector	GTCT	CTGA	(Celinska et al. 2017)
2	JME4366	sgRNA::RFP	sgRNAplatform site	CTGA	AGGT	This study
3a	GGE105	ylLEU2ex	Excisable *LEU2 Y. lipolytica* marker	AGGT	ACGG	(Celinska et al. 2017)
4	JME4313	ylARS1	Replication in *Y. lipolytica*	ACGG	GCTT	This study
5	GGE152	P2_8UAS TEF	Promoter for Cas9 expression	GCTT	ACAA	(Celinska et al. 2017)
6	JME4311	Cas9-SV40	Codon-optimized Cas9-SV40	ACAA	GTCT	This study
3b	GGE216	ylURA3ex	Excisable *URA3 Y. lipolytica* marker	AGGT	ACGG	(Fickers et al. 2003)
3c	GGE176	ylLYS5ex	Excisable *LYS5 Y. lipolytica* marker	AGGT	ACGG	(Celinska et al. 2017)
3d	GGE367	HPHex	Hygromycin B resistance	AGGT	ACGG	(Larroude et al. 2019)
3e	GGE368	NATex	Nourseothricin resistance	AGGT	ACGG	(Larroude et al. 2019)
3f	GGE268	EYK1ex	Growth on erythritol	AGGT	ACGG	This study

The acceptor vectors were assembled using the Golden Gate method in accordance with the Larroude et al. protocol (Larroude et al. 2019). Plasmid DNA was extracted using a commercial miniprep kit (NucleoSpin® Plasmid, Macherey–Nagel) in accordance with the manufacturer’s instructions. Correct assembly was verified by colony PCR and by plasmid digestion with the BglII restriction enzyme. To build the different acceptor vectors, alternative versions of fragment 3 with the different *Y. lipolytica* markers were employed (from 3a to 3f, see Table 1).

Digestion of the acceptor vectors with BsmBI released the RFP gene and allowed the 20-nt target sequence to be inserted at that position. This process made it possible to rapidly visually screen for positive *E. coli* clones that had correctly assembled the CRISPR/Cas9 vectors, in which the gRNA had replaced the RFP gene.

### gRNA design and plasmid construction

The gRNA was designed with the CRISPOR tool (https://crispor.tefor.net/). We chose target sequences with high efficiency scores and low numbers of predicted off-target sites; our preference was for target sequences in the middle of the open reading frame. The target sequences were 20 nt in length and did not include the PAM sequence. The gRNA was introduced into the acceptor vectors by annealing two overlapping oligonucleotides that generated overhangs matching those of the BsmBI site of the acceptor vectors. The oligonucleotides had the following structure: forward oligonucleotide: 5′TTCGATTCCGGGTCGGCGCAGGTTGNNNNNNNNNNNNNNNNNNNNGTTTTA 3′ reverse oligonucleotide: 5′GCTCTAAAACNNNNNNNNNNNNNNNNNNNNCAACCTGCGCCGACCCGGAAT 3′, where N represents the 20-nt target sequence. The underlined letters indicate the 4-nt overhang. The complementary gRNA oligonucleotides were phosphorylated and annealed as follows: we mixed 1 μL T4 Kinase (New England Biolabs, Ipswich, MA), 1 μL forward oligonucleotide (100 μM), 1 μL reverse oligonucleotide (100 μM), 1 μL T4 Ligase Buffer (10X; New England Biolabs), and 6 μL H_2_O; we incubated the mixture at 37 °C for 30 min and then heated the mixture so it stayed at 95 °C for 5 min; and we allowed the temperature to drop back down to 25 °C at a rate of 5 °C min^−1^. The reaction mixture was diluted with water (ratio of 1:200) before being assembled with the CRISPR acceptor vector.

The gRNA double-stranded insert was assembled with the pGGA_CRISPRyl acceptor vector via the Golden Gate method as follows: we mixed 2 μL gRNA insert, 100 ng pGGA_CRISPRyl, 2 μL T4 Ligase Buffer (New England Biolabs), 1 μL BsmBI (New England Biolabs), 1 μL T7 Ligase (New England Biolabs), and 20 µl H_2_O, and we incubated the mixture in a thermocycler using the following program: [5 min at 55 °C, 5 min at 16 °C] × 30, 5 min at 50 °C, and 5 min at 80 °C. We used 10 μL of the assembly mix to transform *E. coli* and then grew the bacteria on LB ampicillin plates. Plasmid DNA was extracted using a commercial miniprep kit (NucleoSpin® Plasmid, Macherey–Nagel) in accordance with the manufacturer’s instructions. Correct assembly of the positive transformants (white *E. coli*) was verified either by colony PCR or by plasmid digestion with the BglII restriction enzyme. The status of positive clones was confirmed via sequencing.

### Transformation of the pGGA_CRISPRyl-gRNA plasmids to perform gene editing in *Y. lipolytica*

Transformation of *Y. lipolytica* was performed using the lithium-acetate method (Barth and Gaillardin 1996). Cells were either plated directly onto selective media for the direct selection of transformants (no outgrowth step), or an outgrowth step was performed on selective liquid media and allowed recovery. In the outgrowth step, cells were cultured in 5 mL of selective media for 24 h and then diluted to obtain 50–100 colonies per plate of rich YPD medium. The transformants could then be tested for the desired phenotype. The gene disruption success rate was determined by counting the number of colonies that showed the expected phenotype; the status of positive clones was confirmed via sequencing. After the screening step, the clones with the desired phenotype were grown in rich liquid YPD medium for 12–24 h to cure the CRISPR\Cas9 plasmid.

### Transformation using the pGGA_CRISPRyl-gRNA plasmids and the deletion cassette for specific locus integration

The deletion cassette was composed of a *URA3*ex expression cassette flanked upstream and downstream by the homologous recombination sequences for the *GSY1* gene. Overall structure of the cassette and construction has been described elsewhere (Fickers et al. 2003). *Y. lipolytica* was transformed using the lithium-acetate method (Barth and Gaillardin 1996). Then, 500 ng of the CRISPR-Cas9 vector was co-transformed with 500 ng of the NotI-digested deletion cassette. The transformation reaction was inoculated in 9 mL of selective liquid medium and cultured for 48 h. One ml of the culture was then transferred into YPD medium for 24 h to allow plasmid curing. Finally, the culture was diluted and plated so as to obtain 50–100 colonies per plate.

When the deletion cassette was transformed without the CRISPR\Cas9 vector, no outgrowth was performed, and 200 µL of the transformation reaction was directly grown on plates containing selective agar.

### Mutant phenotype analysis

After the transformation with the CRISPR\Cas9 vector, the outgrowth step, and the plating, the colonies could undergo phenotype screening.

#### *gsy1* disruption

When *gsy1* had been successfully disrupted, the phenotype was an absence of glycogen accumulation, which could be visualized using staining with Lugol’s iodine (prepared by mixing water solutions of 2% KI and 1% I_2_ at a ratio of 1:1). Colonies in which *gsy1* had been successfully disrupted remained clear, while colonies containing active GSY1 were brown. There are two screening options. The clones can be tested on their plates, by adding 4 mL of Lugol’s iodine. They can also be tested in 96-well microplates after 24 h of culture on YPD medium: 30 µL of Lugol’s iodine is added to each well after the supernatant culture has been eliminated.

#### *eyk1* and *eyd1* disruption

Transformants were grown on both YPD and YNB-erythritol-lysine media for 48 h at 28 °C. Then, we assessed whether clones were able to use erythritol as a carbon source. When a clone grew on YPD but not on YNB-erythritol-lysine, it indicated that *eyk1* or *eyd1* had been disrupted. The JMY7126 strain (*Δeyk1*) was used as the positive control.

#### *mfe1* disruption

Clones in which *mfe1* had been disrupted should be unable to use oleic acid as a carbon source. Transformants were grown on both YPD and YNB-oleic acid media for 48 h at 28 °C. When a clone grew on YPD medium but not on YNB-oleic acid medium, it indicated that *mfe1* had been disrupted. The strain JMY1888 (*Δmfe1*) was used as a positive control.

#### *ura3* disruption

Transformants were grown on both YPD medium and YNB medium without uracil for 48 h at 28 °C. When a clone grew on YPD but not on YNB without uracil, it indicated that *ura3* had been disrupted.

#### *lip2* disruption

The *LIP2* gene encodes the main extracellular lipase, so Δ*lip2* strains display a reduced halo of triglyceride hydrolysis on YNB medium containing tributyrin (Pignede et al. 2000). Transformants were grown on YNB medium containing tributyrin for 48 h at 28 °C. Clones with strongly reduced halos were considered to carry a disrupted version of *LIP2*.

### Colony PCR and sequence-based verification of gene disruption

For positive clones, we verified gene disruption via sequencing. We employed specific primers that spanned the gRNA target regions (supplementary Table S2). First, the regions were amplified by colony PCR. Single colonies were picked with a tip and transferred to 2µL water and cells were lysed with a 15 min heating cycle in the thermocycler. The cells lysed were directly used in the standard PCR reaction. The amplified fragments were then purified using gel extraction and PCR purification kits (NucleoSpin® Gel and PCR Clean-Up, Macherey–Nagel) and sequenced. The sequences were aligned against reference sequences to identify the mutations in the target sequence.

## Results

### Construction of a versatile CRISPR/Cas9 vector set

To extend the set of synthetic biology tools available for *Y. lipolytica*, we wished to develop a systematic backbone method for building a set of CRISPR/Cas9 vectors. Our design was based on the basal structure of the plasmid developed by Schwartz et al. (Schwartz et al. 2016), and the Golden Gate method made it possible to assemble and switch out different parts. We took advantage of a large set of Golden Gate bricks that were recently made available for *Y. lipolytica* (Larroude et al. 2019). For our specific CRISPR/Cas9 vector set, the necessary parts include i) a bacterial plasmid containing the ampicillin resistance gene and the ColE1 region for selection and propagation in *E. coli*; ii) a *Y. lipolytica* selection marker; iii) the ARS/CEN fragment allowing plasmid replication in *Y. lipolytica*; iv) a *Y. lipolytica* promoter for endonuclease expression; v) the Cas9 endonuclease optimized for *Y. lipolytica*; and vi) a region for cloning gRNA. In association with the latter, we implemented a direct screening method for verifying that the recombinant plasmid had incorporated the gRNA: we integrated a RFP chromophore gene at the gRNA cloning site that would be later released, allowing the assembly of the 20-nt target sequence at that same location (Celinska et al. 2017). The set of bricks designed and used to assemble the CRISPR/Cas9 acceptor vectors are listed in Table 1. These acceptor vectors were first put together using BsaI overhang sequences, as described in Larroude et *al.* (Larroude et al. 2019). These acceptor vectors were then ready to be assembled with the gRNA fragment of choice using the BsmBI overhang sequences, as described in the Material and Methods. The overall design is depicted in Fig. 1; the Golden Gate overhang sequences, the BsaI overhang sequences for backbone construction, and the BsmBI overhang sequences for gRNA cloning are indicated.

Using the pool of Golden Gate bricks, we built a set of acceptor vectors that were ready for immediate use in gRNA cloning employing different markers. We used LEU2ex, URA3ex, and LYS5ex for the auxotrophic markers; NATex and HPHex for the antibiotic markers; and EYK1ex for the CSM markers. All the acceptor vectors are listed in Table 2.

**Table 2:** List of the gRNA acceptor vectors that were built

Strain number	Vector name	Marker
JME4390	GGA_ LEU2ex_CrisprCas9-yl_RFP	LEU2ex
JME4393	GGA_ LYS5ex_CrisprCas9-yl_RFP	LYS5ex
JME4472	GGA_URA3ex_CrisprCas9-yl_RFP	URA3ex
JME4580	GGA_HPHex_CrisprCas9-yl_RFP	Hygromycin optimized for *Y. lipolytica*
JME4599	GGA_NATex_CrisprCas9-yl_RFP	Nourseotricin optimized for *Y. lipolytica*
JME5000	GGA_EYK1ex_CrisprCas9-yl_RFP	EYK1ex

### Validation of genome editing

We tested our CRISPR/Cas9 system by first targeting genes that can be used as markers: *EYK1*, *EYD1*, and *URA3*. The *EYK1* gene encodes erythrulose kinase, which is involved in erythritol catabolism. The disruption of this gene allows the selection of strains that cannot use erythritol as their sole carbon source (Carly et al. 2017). The *EYD1* gene encodes erythritol dehydrogenase, which is also involved in erythritol catabolism (Carly et al. 2018). The *URA3* gene is involved in uracil metabolism. The disruption of *EYK1* and *EYD1* increased the strength of recently developed erythritol-inducible promoters pEYK1 and pEYD1 (Trassaert et al. 2017; Park et al. 2019). It is therefore particularly useful to disrupt these genes in strains that contain expression cassettes based on these promoters.

We then extended our testing to include genes related to lipid metabolism: *MFE2*, *GSY1*, and *LIP2*. The *MFE2* gene encodes the multifunctional enzyme, which is involved in the fatty acid degradation pathway. Its inactivation results in cells that are unable to use fatty acids as their sole carbon source (Smith et al. 2000). The *GSY1* gene encodes glycogen synthase, which is involved in glycogen synthesis. Its inactivation results in carbon storage being redirected from sugars (glycogen) to lipids (triacyl glycerol) (Bhutada et al. 2017). The *LIP2* gene encodes lipase, which is involved in external lipid degradation (Pignede et al. 2000).

gRNAs were designed for all these targets and then assembled in the CRISPR/Cas9 vector JME4390. All gene editing was performed in the strain Po1d (JMY195). Table 3 shows the editing success rate (i.e., following phenotype screening and sequence-based verification; see the Materials and Methods section). Editing success was highly variable (7–70%), and it depended on the target gene. It was also dependent on the gRNA sequence (data not shown), a finding reported in a large scale study in *Y. lipolytica* (Schwartz et al. 2019). Because it was easy to screen and had a median editing success rate, we chose to target *GSY1* using our CRISPR/Cas9 vector set. More specifically, the CRISPR/Cas9-gGSY1 vectors were used in different genetic backgrounds adapted to the six markers (Table 4). The results show that editing success was highly dependent on the marker/strain combination used and ranged from 4% (JMY195 with the HPHex marker) to 81% (JMY330 with the LEU2ex marker and JMY195 with the NATex marker). For one marker, EYK1ex, no positive clones were obtained (Table 4). For this strain/marker combination, either editing failed completely or the success rate was below 1% and thus undetectable.

**Table 3:** Target gene, ID, editing success rate, and the number of clones tested using the backbone vector JME4390 in the JMY195 strain

Gene	YALI ID	Editing success rate (number of clones tested)
MFE2	YALI0E15378g	7% (30)
EYK1	YALI0F01606g	17% (30)
EYD1	YALI0F01650g	7% (28)
GSY1	YALI0F18502g	20% (48)
URA3	YALI0E26741g	20% (48)
LIP2	YALI0A20350g	70% (30)

**Table 4:** Editing success rate for the *GSY1* gene and the number of clones tested for the different markers and strains

Vector	Marker	Strain	Editing success rate (%)	Number of transformants tested
JME4473	URA3ex	Y2033	19	48
JME4473	URA3ex	Y195	21	48
JME4392	LEU2ex	Y195	46	96
JME4392	LEU2ex	Y330	81	64
JME4425	LYS5ex	Y5211	56	96
JME4600	NATex	Y195	81	32
JME4600	NATex	Y330	37	80
JME4600	NATex	WT5	56	48
JME4600	NATex	W29	57.5	40
JME4759	HPHex	W29	45	13
JME4759	HPHex	Y195	4	48
JME5019	EYK1ex	Y7123	0	48

In *Y. lipolytica*, homologous recombination is not very efficient. To confirm that our set of CRISPR/Cas9 vectors could help integrate DNA via homologous recombination, we compared the rate of homologous recombination with and without co-transformation by CRISPR/Cas9 vectors in a standard genetic background and in a *Y. lipolytica* strain deleted for *ku70*, which has shown improved homologous recombination (Verbeke et al. 2013). A classical disruption cassette composed of a URA3 expression cassette flanked by 1-kb homologous regions upstream and downstream from the *GSY1* gene—was used to transform Po1d and Po1d *Δku70*. Table 5 shows that using a CRISPR/Cas9 vector in tandem with the integration cassette strongly improved homologous recombination. The success rate was nearly 100% in the *Δku70* background and 83% in the Po1d background. When the cassette alone was used, the success rate was only around 15%. High success rates were obtained in a similar experiment using the same backbone plasmid (Schwartz et al. 2016). The low level of homologous recombination in *Y. lipolytica* is a drawback in standard deletion and targeted integration procedures. Moreover, the need for a long flanking region complicates cloning, but reducing the size of the homologous region strongly reduces the rate of homologous recombination. To determine if using a CRISPR/Cas9 vector in tandem with a shortened homologous region could improve the rate of correct integration, similar experiments were performed using homologous regions of different lengths (100 bp and 50 bp) located both upstream and downstream from the *GSY1* gene. When the 100-bp region was used in the absence of the CRISPR/Cas9 vector, no positive clones resulted from the deletion of the *GSY1* gene (Table 5). However, when the CRISPR/Cas9 vector was employed, the success rate was reasonable (25%); it was very high (88%) in the *Δku70* background. When the 50-bp region was used, there were no positive clones (Table 5). In short, using a CRISPR/Cas9 vector makes it possible to strongly reduce the size of the homologous recombination region (down to 100 bp) and still obtain a reasonable rate of successful insertion with or without using a *Δku70* background.

**Table 5:** Rate at which the *GSY1* gene was successfully edited and the number of clones tested in different genetic contexts employing homologous recombination regions of different lengths

	1-kb homologous flanking region		100-bp homologous flanking region		50-bp homologous flanking region	
	Number of positive clones	Editing success rate (%)	Number of positive clones	Editing success rate (%)	Number of positive clones	Editing success rate (%)
CRISPRgsy1	10/48^*^	20^*^	N/A	N/A	N/A	N/A
HR (PUTgsy1)	7/48	15	0/48	0	0/48	0
CRISPRgsy1 + HR (PUTgsy1)	40/48	83	12/48	25	0/48	0
CRISPRgsy1 + HR (PUTgsy1) + delta KU	48/48	100	42/48	88	0/48	0

^*^In this case no disruption cassette was used, only the efficiency of the CRISPR-Cas9 system was evaluated as a control

*N/A* Not applicable

One of the advantages of having access to a large set of markers is that it becomes faster to carry out multiple deletions in a single strain. We therefore wished to verify that we could edit multiple genome locations by transforming yeast using two CRISPR/Cas9 plasmids in tandem. In the strain JMY5217 (Po1d Lys^−^Leu^−^), we simultaneously disrupted *gsy1* and *ura3* using the JME 4392 (CrCas9-Leu2-gGSY) JME 4453 (CrCas9-Lys5-gURA), respectively. For the 48 colonies tested, the success rate was 50% for *ura3* and 85% for *gsy1*. All the *ura3* knockouts were also *gsy1* knockouts. When the markers were inversed, by using JME4425 (CrCas9-Lys5-gGSY) and JME 4452 (CrCas9-Leu2-gURA), our success rate was nil for *ura3* and 62% for *gsy1*. These results show that, in *Y. lipolytica*, it is possible to carry out simultaneous transformation employing multiple CRISPR/Cas9 vectors that have the same backbone but that express different markers. However, some gRNA-marker combinations appear less efficient than others as already observed (Table 4). In particular, Leu2-gURA was not successful in our hand in the JMY5217 background while it is in the JMY195 background (Table 3).

### Genome editing in wild-type strains using CRISPR/Cas9 vectors

**Fig. 2:**
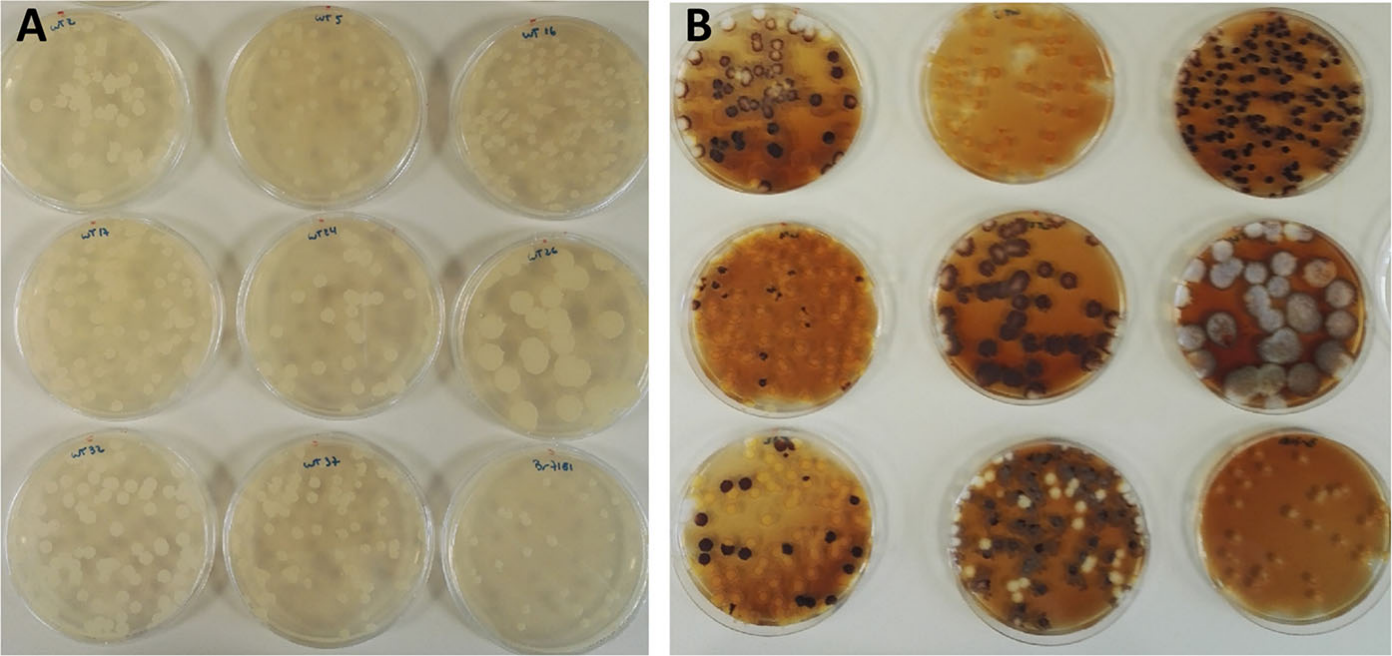
CRISPR/Cas9-mediated disruption of the *GSY1* gene in wild-type strains. **a** Clones on plates before Lugol staining. **b** Clones on plates after Lugol staining. List of strains tested (from top left to bottom right): CBS 2074, CBS 6125, CLIB 791, CLIB 879, DBVPG 4400, DBVPG 5851, NCYC 3271, PYCC 4743 and IMUFRJ 50682

Most of the genetic engineering that takes place in *Y. lipolytica* involves a small subset of laboratory strains that have been specifically developed for this purpose by introducing auxotrophies. Because standard engineering methods therefore rely on auxotrophies, limited use has been made of the diverse characteristics displayed by different *Y. lipolytica* wild-type strains. However, as mentioned in the introduction, wild-type strains can outperform laboratory strains. They are therefore a better chassis for carrying out further modifications. One of our objectives in developing this set of CRISPR/Cas9 vectors with dominant markers was to be able to perform genome editing in wild-type *Y. lipolytica* strains. We used our CRISPR/Cas9-hph vector, into which *gsy1* gRNA had been introduced, to transform a collection of wild-type strains as a proof of principle. This plasmid was used to transform nine different wild-type strains representing a broad range of origins (see Supplementary Table S1). The rate at which we successfully disrupted *gsy1* was determined using Lugol staining, as shown in Fig. 2 (A and B); the editing success rates are indicated in Table 6. Positive clones were sequenced to confirm editing success.

**Table 6:** Number of clones with and without the clear colony phenotype, number of positive clones sequenced, and editing success rate

Strain	Colonies with clear phenotype/total clones	Positive clones/total clones sequenced	Editing success rate (%)
IMUFRJ 50682	48/48	3/3	100
CBS 2074	9/48	1/1	18.75
CBS 6125	48/48	3/3	100
CLIB 879	90/105	3/3	85
DBVPG 5851	1/26	1/1	3.8
NCYC 3271	42/48	3/3	87.5
PYCC 4743	18/48	1/1	37.5
CLIB 791	0/153	0	0
DBVPG 4400	0/46	0	0

The editing success rate was determined based on the staining patterns of the transformants (dark vs. clear in response to Lugol staining). As variability in staining patterns was observed among strains (Fig. 2), we sequenced between one and three clear clones for each strain to validate the genome editing process. In all cases, we observed that genome editing occurred upstream from the PAM sequence, at the gRNA target sequence. In general, our CRISPR/Cas9 vectors with dominant markers were well suited to genome editing in wild-type strains. All the wild-type *GSY1* genes were sequenced, and their sequences were compared to the CLIB122 sequence that was used to design the gRNA. No differences were seen in the gRNA target region, excluding potential mismatch bias. We observed dramatic differences in editing success: it ranged from 100% for the strains IMUFRJ 50682 and CBS 6125 to 0% for strains CLIB 791 and DBVPG 4400 (Table 6). This observation implies that it might be easier to genetically engineer some wild-type strains than others, but wild-type strains could still outperform some of the more widely used laboratory strains. We were able to easily transform all the wild-type strains with our vector even if two of them failed to show any signs of editing. All the strains tested are sensitive to hygromycin (growth inhibition between 60 and 80 µg/mL) and no major differences in sensitivity were observed that could explained differences in transformability and/or editing.

## Discussion

Here, we describe how we used the Golden Gate method to assemble a set of CRISPR/Cas9 vectors, each containing a different marker, that can be employed to perform genome editing in *Y. lipolytica*. The vectors are now available via Addgene (129,656–129,661). In all these vectors, any gRNA sequence can be easily and rapidly introduced using Golden Gate assembly.

We built our system by taking advantage of the large set of bricks available for *Y. lipolytica* and a recently published Golden Gate protocol (Larroude et al. 2019). In our system, the Cas9 endonuclease could be placed under the control of different promoters that vary in strength and inducibility, which could be useful to temporally control expression. We decided to employ the 8UASpTEF promoter because our comparison of the results obtained with pTEF and 8UASpTEF found no significant differences in editing efficiency (data not shown). Borsenberger et al*.* showed that promoter strength was not critical and that pTEF provides adequate results compared to 8UASpTEF when it comes to the expression of the Cas9 endonuclease in *Y. lipolytica* (Borsenberger et al. 2018). The only constraint is that RNA structure must be compatible. Egermeier et al. (Egermeier et al. 2019) recently published a Golden Gate protocol for building *Y. lipolytica* CRISPR/Cas9 vectors, but its applicability is more limited than that of our protocol because it does not include a range of markers or a rapid-screening method (i.e., we used RFP as a negative reporter of assembly success). They were able to knockout *LEU2* in a wild-type strain and showed that their system was functional (editing success rate: 6–25%). They used a different CRISPR/Cas9 system that is based on the HH ribozyme and a humanized Cas9 that is not codon optimized for *Y. lipolytica* (Gao et al. 2016). Systems utilizing HH ribozyme gRNA processing have been shown to be less efficient than systems utilizing the PolIII promoter system (Schwartz et al. 2016); we used the latter system here, and it was coupled with a *Y. lipolytica* optimized Cas9. Accordingly, in *Y. lipolytica*, codon-optimized Cas9 is expressed at higher levels than the humanized Cas9 when the polIII system is employed (Borsenberger et al. 2018).

We also used CRISPR/Cas9-mediated cutting to introduce an expression cassette at a specific locus. The editing success rate was much higher with CRISPR/Cas9 than without CRISPR/Cas9, and we were able to drastically reduce the length of the homologous recombination region without having to use a Δ*ku* background. This approach allowed us to avoid the pitfalls associated with the Δ*ku* background. It can also simplify and speed up metabolic engineering because integrating a large pathway at a specific locus can sometimes be a difficult task (Schwartz et al. 2017b), and using a marker can drastically improve efficiency. Marker-free integration, in which Cas9 cuts are repaired via homologous recombination, can work in *Y. lipolytica*, but the editing success rate is not consistent outside of the Δ*ku* background (Gao et al. 2016; Schwartz et al. 2017b; Holkenbrink et al. 2018).

All the vectors that we built were tested using different targets, and genome editing was successful in all cases but one (the EYK1ex marker). We took advantage of antibiotic markers to exploit the natural diversity of *Y. lipolytica* and validated our CRISPR/Cas9 vectors in a large number of wild-type strains. The editing success rate for *gsy1* disruption differed dramatically among strains even though they harboured an identical *gsy1* sequence. The colony morphology are diverse (Fig. 2) and may reflect physiology differences between strains. This can affect the rate of transformants if, for example, cell wall structures are different. Transformation procedure is the one setup and optimized for the laboratory strains W29 or CLIB122. Physiology differences may require different optimization for the other wild-type strains, which could improve transformation efficiency and ultimately editing efficiency. The phenotype screening also highlighted differences in staining patterns that probably reflect differences in glycogen accumulation. In *Y. lipolytica*, glycogen storage is detrimental to lipid accumulation (Bhutada et al. 2017), so these results indicate that *gsy1* disruption (dark-stained strains) may have a great impact on lipid production. Another, less likely, hypothesis is that the penetration of Lugol’s iodine varied among strains.

To our knowledge, our set of CRISPR/Cas9 vectors is the most extensive to date for carrying out genome editing in *Y. lipolytica*.

## Electronic supplementary material

Below is the link to the electronic supplementary material.

Supplementary file1 (DOCX 25 kb)
