# Supplementary material for: A set of Yarrowia lipolytica CRISPR/Cas9 vectors for exploiting wild-type strain diversity
Source: Biotechnol Lett. 2020 Jan 23;42(5):773–85. doi: 10.1007/s10529-020-02805-4 (PMC7101291; doi:10.1007/s10529-020-02805-4)
Supplement: Supplementary file 1 — Supplementary file1 (DOCX 25 kb) [file 10529_2020_2805_MOESM1_ESM.docx]

## supplementary data

**Supplementary Table S1.** *E. coli* and *Y. lipolytica* strains and plasmids used in this study

| Strain (host strain) | Plasmid, genotype | References |
| --- | --- | --- |
| ***E. coli* strains** |  |  |
| DH5**α** | Φ80*lacZ*Δm15 Δ(*lac*ZYA-*arg*F) U169 *rec*A1 *end*A1 *hsd*R17 (r_k_^−^, m_k_^+^) *pho*A *sup*E44 *thi*-1 *gyr*A96 *rel*A1 λ^-^ | (Promega) |
| GGE029 | GGE pSB1A3-GB3 | (Celinska et al. 2017) |
| JME4315 | pGM_sgRNA platform | This work |
| JME4473 | p8UASTEF_CRISPR-Cas9_URA3ex_gGSY, AmpR | This work |
| JME4392 | p8UASTEF_CRISPR-Cas9_LEU2ex_gGSY, AmpR | This work |
| JME4425 | p8UASTEF_CRISPR-Cas9_LYS5ex_gGSY, AmpR | This work |
| JME4600 | p8UASTEF_CRISPR-Cas9_NATex_gGSY, AmpR | This work |
| JME4759 | p8UASTEF_CRISPR-Cas9_HPHex_gGSY, AmpR | This work |
| JME5019 | p8UASTEF_CRISPR-Cas9_EYKex_gGSY, AmpR | This work |
| JME4452 | p8UASTEF_CRISPR-Cas9_LEU2ex_gURA, AmpR | This work |
| JME4453 | p8UASTEF_CRISPR-Cas9_LYS5ex_gURA, AmpR | This work |
| ***Y. lipolytica* strains** |  |  |
| W29 | *Wild type* | (Barth and Gaillardin 1996) |
| Po1d, JMY195 | *MATA* *leu2*-*270* *ura3*-*302* *xpr2*-*322* | (Barth and Gaillardin 1996) |
| JMY330 | *MATA leu*2‐*270* *xpr*2‐*322* | (Haddouche et al. 2010) |
| JMY2033 | Po1d*, ura3::LEU2ex-zeta* | (Lazar et al. 2013) |
| JMY2394 | Po1d *Δku70* | (Verbeke *et al.* 2013) |
| JMY5211 | JMY2033 *lys5::URA3ex* | Unpublished |
| JMY5217 | Po1d *lys5::URA3ex* | Unpublished |
| JMY7123 | Po1d, Δ*lip2* Δ*lip7* Δ*lip8* ura3::LEU2-ZETA *lys5*::*URA3*ex Δ*eyk1* | Soudier *et al. to be published* |
| JMY7126 | Po1d, Δ*lip2* Δ*lip7* Δ*lip8* *ura3*::*LEU2*-ZETA Δ*lys5* Δ*eyk1* | Soudier *et al. to be published* |
| JMY7923 | *Wild-type* CBS 2074*,* Isolated from olives. |  |
| JMY7926 | *Wild-type* CBS 6125. Isolated from maize-processing plant. |  |
| JMY7936 | *Wild-type* CLIB 791. Isolated from goat’s cheese |  |
| JMY7937 | *Wild-type* CLIB 879*.* Isolated from cheese. |  |
| JMY7941 | *Wild-type* DBVPG 4400*.* Isolated from commercial compost. |  |
| JMY7942 | *Wild-type* DBVPG 5851*.* Isolated from soil of palm tree. |  |
| JMY7945 | *Wild-type* NCYC 3271. Isolated from sugar cane peels. |  |
| JMY7950 | *Wild-type* PYCC 4743*.* Isolated from see water. |  |
| JMY7181 | *Wild-type.* IMUFRJ 50682 Isolated from estuary. | (Nunes et al. 2013) |

**Supplementary Table S2. Primers list**

| Primer name | Sequence | Use |
| --- | --- | --- |
| GGP_E.coliVector_N_Fw | gcGGTCTCtGTCTtcttcctgcagtccggcaaaaaag | Amplification of *E. coli* part for constructing Cr Vector |
| GGP_E.coliVector_O_Rv | gcGGTCTCtTCAGaggctcttcgaattccagaaatcatc | Amplification of *E. coli* part for constructing Cr Vector |
| RFP-SfiI-Fw | ccGGCCATCTGGGCCctagagcaatacgcaaaccgc | Amplification of RFP for cloning into gRNA Platform |
| RFP_SfiI-Rv | ccGGCCCAGATGGCCtatataaacgcagaaaggcccac | Amplification of RFP for cloning into gRNA Platform |
| GSY_sgRNA_Fw | gggCGTCTCGtTTCGATTCCGGGTCGGCGCAGGTTGGCTGTTCGAGGTCGCCACCG | sgGSY cloning |
| GSY_sgRNA_Rv | cccCGTCTCtAGCTCTAAAACCGGTGGCGACCTCGAACAGCCAACCTGCGC | sgGSY cloning |
| T7_GSY_Fw | TATGGGTACAGGACGTGCATAG | GSY screening |
| T7_GSY_Rv | AGAGTAGCGTGTGTGGTGAAGA | GSY screening |
| URA_sgRNA_Fw | TTCGATTCCGGGTCGGCGCAGGTTGgAAGGAACTTGCTCTTAAGCAGTTTTA | sgURA cloning |
| URA_sgRNA_Rv | GCTCTAAAACTGCTTAAGAGCAAGTTCCTTcCAACCTGCGCCGACCCGGAAT | sgURA cloning |
| T7_URAorf_Fw | ATGCCCTCCTACGAAGCTCGAG | URA screening |
| T7_URAorf_Rv | GTTCTGGCCGTACAGACCTC | URA screening |
| Lip2_sgRNA_Fw | TTCGATTCCGGGTCGGCGCAGGTTGgATCTTCAAGCCCTTCAACTGGTTTTA | sgLip2 cloning |
| Lip2_sgRNA_Rv | GCTCTAAAACCAGTTGAAGGGCTTGAAGATcCAACCTGCGCCGACCCGGAAT | sgLip2 cloning |
| T7_Lip_Fw | CTTTCCACCATCCTCTTCACAG | Lip2 screening |
| T7_Lip_Rv | CTCCTGGCCAAAGAAGAGTTTA | Lip2 screening |
| gRNA1-MFE2-Fwd | TTCGATTCCGGGTCGGCGCAGGTTGGGACAAGGTTACCCGAGCTGCCGTTTTA | sgMFE2 cloning |
| gRNA1-MFE2-Rev | GCTCTAAAACGGCAGCTCGGGTAACCTTGTCCAACCTGCGCCGACCCGGAAT | sgMFE2 cloning |
| ForT7_MFE _Endon-Test-Fw-MFE | GTCGACGAGATTGTTTCCAAG | MFE2 screening |
| ForT7_MFE_Endon-Test-Rv-MFE | GGTCGCTTAACAGACTCCTCAG | MFE2 screening |
| gRNA-EYK1-Fwd | TTCGATTCCGGGTCGGCGCAGGTTGGCAAGCTCGTGCCCTCCGACAGTTTTA | sgEYK1 cloning |
| gRNA-EYK1-Rev | GCTCTAAAACTGTCGGAGGGCACGAGCTTGCAACCTGCGCCGACCCGGAAT | sgEYK1 cloning |
| T7_ EYK1_Fw | ATCTGTTCAACGAGACTGACGA | EYK1 screening |
| T7_EYK1_Rv | CCTGAACTTTACGAAGGATCG | EYK1 screening |
| ht17-gRNA-EYD1-Fwd | TTCGATTCCGGGTCGGCGCAGGTTGGTCAGTCTGCCTCGCCGAGCTGTTTTA | sgEYD1 cloning |
| ht18-gRNA-EYD1-Rev | GCTCTAAAACAGCTCGGCGAGGCAGACTGACCAACCTGCGCCAACCCGGAAT | sgEYD1 cloning |
| ht35-Verif-EYD1-Fwd | GCCCATCTCGGCACCCTACA | EYD1 screening |
| ht36-Verif-EYD1-Rev | GGCTCAAACTGGCCGAGCTC | EYD1 screening |

Barth G, Gaillardin C (1996) Yarrowia lipolytica. In: Wolf K, Breuning KD, Barth J (eds) Non-conventional Yeasts in Biotechnology. Springer-Verlag, Berlin, pp 313-388

Celinska E, Ledesma-Amaro R, Larroude M, Rossignol T, Pauthenier C, Nicaud JM (2017) Golden Gate Assembly system dedicated to complex pathway manipulation in Yarrowia lipolytica Microb Biotechnol 10:450-455 doi:10.1111/1751-7915.12605

Haddouche R, Delessert S, Sabirova J, Neuvéglise C, Poirier Y, Nicaud J-M (2010) Roles of multiple acyl-CoA oxidases in the routing of carbon flow towards β-oxidation and polyhydroxyalkanoate biosynthesis in *Yarrowia lipolytica* FEMS Yeast Res 10:917-927 doi:10.1111/j.1567-1364.2010.00670.x

Lazar Z, Rossignol T, Verbeke J, Crutz-Le Coq A-M, Nicaud J-M, Robak M (2013) Optimized invertase expression and secretion cassette for improving *Yarrowia lipolytica* growth on sucrose for industrial applications J Ind Microbiol Biotechnol 40:1273-1283 doi:10.1007/s10295-013-1323-1

Nunes PMB, da Rocha SM, Amaral PFF, da Rocha-Leão MHM (2013) Study of trans–trans farnesol effect on hyphae formation by Yarrowia lipolytica Bioprocess Biosystems Eng 36:1967-1975 doi:10.1007/s00449-013-0973-8

Verbeke J, Beopoulos A, Nicaud J-M (2013) Efficient homologous recombination with short length flanking fragments in Ku70 deficient Yarrowia lipolytica strains Biotechnol Lett 35:571-576 doi:10.1007/s10529-012-1107-0
